# Antlerogenic stem cells extract accelerate chronic wound healing: a preliminary study

**DOI:** 10.1186/s12906-021-03336-9

**Published:** 2021-05-29

**Authors:** Janusz Kmiecik, Michał Jerzy Kulus, Jarosław Popiel, Agnieszka Cekiera, Marek Cegielski

**Affiliations:** 1 “Zdrowie i Uroda” Janusz Kmiecik Doctor’s Office, Krotoszyn, Poland; 2 Department of Ultrastructural Research, Wroclaw Medical University, Ul. Chałubińskiego 6a, 50-368 Wrocław, Poland; 3 Department of Internal Medicine and Clinic of Diseases of Horses, Dogs and Cats, Wrocław University of Environmental and Life Sciences, Wrocław, Poland

**Keywords:** Chronic wounds, Stem cells therapy, Antlerogenic stem cells

## Abstract

**Background:**

Chronic wounds constitute a significant medical and social problem. Chronic wound treatment may be supported by various techniques, such as negative pressure therapy, phototherapy or stem cells therapy, yet most of those supporting therapies need more evidence to be used for standard wound care. Current study covers the use of sonicated Antlerogenic Stem Cells (ASC) extract on chronic wounds.

**Methods:**

Study was performed on 20 dermatological patients with venous leg ulcers, divided into two groups – treated with and without ASC extract respectively. The area and circumference of the wounds during the follow-up visits were measured on the wound imprint. Dynamics of wound healing was determined and compared between control and study group; statistics includes changes in absolute values (wound area, circumference), as well as relative (percentage of wound decrease, circumference/area ratio) and their change in time. For the purpose of Ki-67 immunohistochemical staining, sections were sampled from the wound edge at distinct check-points during therapy. Results of both groups were compared with Student test or Mann-Whitney test, depending on results distribution.

**Results:**

Besides Ki-67 expression, all tested wound healing parameters (including relative and absolute wound decrease and changes in circumference/area ratio) were statistically significant more favorable in experimental group.

**Conclusion:**

ASC extract significantly supported standard chronic wound treatment. Due to small population of study the results should be considered preliminary, yet promising for further research.

## Background

Disruption of the integrity of the skin, mucous membranes or organs results in the formation of a wound. They can arise in the course of specific diseases or be the result of physical trauma [1]. Injury results in the initiation of a cascade of processes that lead to its healing. Usually, this wound healing process is divided into four phases: hemostasis, inflammation, proliferation and tissue remodeling [2]. Its course may, however, be disturbed by numerous factors leading to an extended healing period. If it exceeds over 12 weeks, it may be referred to as “chronic wounds” [1]. According to another definition, a wound may be considered “chronic” one when it fails to progress in a timely and orderly fashion through the aforementioned healing phases [3].

Chronic wounds is a significant medical and social problem. In developed countries, it is estimated that 1–2% of the population have experienced or will experience chronic wounds in their lifetime [4]. In the United States only, costs directly related to treatment of chronic wounds were estimated at approx. $25 billion in 2007 [5] and at $33 billion in 2014 [6]. It is assumed that this number will continue to rise – due to the increasing life expectancy (as the risk of chronic wounds development increases with age) and the greater incidence of diabetes, obesity, bad nutrition, deep vein thrombosis, chronic diseases and others, which significantly impedes wound healing [6–8]. Moreover, above calculations consider direct costs of treatment only, without taking into account the costs related to loss of productivity or restriction/exclusion from professional activity.

The Wound Healing Society (WHS) distinguishes 4 basic types of chronic wounds: Pressure ulcers, Diabetic ulcers, Venous ulcers and Arterial insufficiency ulcers [9]. Chronic wounds have numerous features that distinguish them from acute wounds – both clinically and biochemically. While in the case of acute wounds, the treatment phases mentioned above occur in an organized and correct manner, in chronic wounds it is prolonged in the inflammation phase and the course of the proliferation or remodeling phase is disrupted or inhibited [10].

The phenotype of cells found in chronic wounds is similar to that of senescent cells; venous ulcer fibroblasts show a worse response to PDGF, a reduced number of TGF-B receptors and inappropriate phosphorylation in key signaling pathways [11]. Synthesis of new Extracellular Matrix (ECM) components is limited, and its organization is disordered [3, 12]. Abundance of inflammatory cells in chronic wounds leads to the increase of Matrix Metalloproteinases (MMPs) that degrade ECM, growth factors and receptors necessary for the synthesis and organization of ECM [13].

Treatment of chronic wounds includes proper debridement, cleansing, keeping moisture balance by proper wound dressing [14]. It may be supported by various techniques, such as negative pressure therapy [15], phototherapy [16] or stem cells therapy [17–19], yet most of those supporting therapies need more evidence to be used for standard wound care [20].

Mesenchymal Stem Cells (MSCs) often used in stem cell-based therapies, are most often obtained from bone marrow (Bone Marrow Derived-MSCs, BM-MSCs) or adipose tissue (Adipose Derived MSCs, ADSCs), although they can also be obtained from the tooth pulp [21, 22]. Such therapies often focus on collecting the target tissue from the patient, culture extracted cells in vitro and use for autogenous transplantation, but commercial lines for allogeneic transplantation are also available [23]. They are implanted either by injection or as cells on bioscaffolds [24].

Products that do not contain viable cells are also used, such as Grafix (Osiris Therapeutics; Columbia, Md.), containing dehydrated and cryopreserved fibroblasts, MSCs and epithelial cells [23]. In this case, regeneration is not assisted by living cells, but rather by proteins and components produced by them.

Current study concerns effectiveness of extract of sonicated Antlerogenic Stem Cells (ASC) on chronic wound in human. Sonicated ASC from MIC-1 cell line [25] were tested on animal models for use in regenerative medicine. Preliminary studies on animals showed promising results [25–29]. However, to the best of our knowledge, current study is the first one testing ASC extract on regeneration in humans.

## Methods

### Antlerogenic stem cells source

The source of ASC in this study was the product: “Revitacell: the preparation for regenerative mesotherapy”. Revitacell contains stem cells homogenate derived from the deer antler stem cell line MIC-1 (listed in CosIng Cosmetic Ingredient Database as: “Deer antler cell extract”) [25]. Full characteristic of MIC-1 cell line is described by Cegielski et al. and Dąbrowska et al. [25, 27].

Homogenate is obtained by sonication of 10^7^cells/ml, harvested from MIC-1 cell culture. Sonication results in plasma membrane rupture and release of cell contents. The final preparation contains a 20% concentration of ASC homogenate with addition of vitamins B5, B3 and allantoin. The final product is a milk-white liquid suspension of watery consistency, packed in 3-ml ampoules. The ingredients of the preparation affect regeneration, hydration and soothe irritation [26].

Revitacell is produced by Stem Cells Spin S.A. laboratories, in accordance with PN-EN ISO 9001:2009, PN-EN ISO 22716:2007 (GMP) and PN-EN ISO 13485:2012.

### Study population

Study included participation of 20 patients from the dermatology clinic „Centrum Zdrowie i Uroda – gabinet Janusz Kmiecik”, Krotoszyn, Poland. All patients suffered from venous leg ulcers and were diagnosed and treated between 2016 and 2019. Patients were divided into two groups (control and experimental), which underwent leg ulcers therapy. Therapy included phototherapy, use of alginate wound dressing sealed with self-adhesive, transparent film dressing (Suprasorb F) and standard procedures for chronic wound treatment [15]. Wound dressing was changed once a day. Both groups were treated equally, yet in experimental group, ASC extract was additionally applied; 1–3 ml (depending on the wound size) were applied on alginate wound dressing, before putting it directly on wound each time the wound dressing was changed. The characteristics of the population of study is presented in Table 1.

**Table 1:** Characteristics of the studied group of patients at the beginning of treatment

Trait	Experimental group Mean (SD)	Control group Mean (SD)
**Age**	67.23 (11.64)	68.57 (11.66)
**Sex**		
Males	5	1
Females	8	6
**Number of wounds/patient**		
**1**	10	6
**2**	2	1
**Comorbidities**		
Diabetes	1	3
Obesity	4	1
Diabetes & obesity	2	1
**Wound area**	22.40 (31.32)	15.69 (24.78)
**Wound circumference**	23.61 (25.38)	14.33 (14.78)

Small sections (2–4 mm in diameter) from the wound edges were cut out with scalpel from each patient during follow-up visits at irregular intervals. The samples were fixed, embedded in paraffin and stored in the form of paraffin blocks which were then used for immunohistochemical analysis. For patients with multiple wounds, only one section was taken during each visit.

During each visit wounds were photographed, wound imprints were taken; Suprasorb F wound dressing was put on the wound and then its circumference was gently outlined with non-toxic pen. Wound imprints were subsequently scanned with scale bar and the area and circumference of the wound were measured with AutoCAD software (Autodesk, USA).

### Wound healing dynamics and statistics

The area and circumference of the wounds during the follow-up visits were measured on the wound imprint (Fig. 2). The dynamics of wound healing was measured by checking how the following parameters changed over time:

- wound area [cm^2^]
- total wound area decrease (calculated as *A*_0_ − *A*_*n*_, where *A*_0_ stands for wound area at the begining of therapy, and *A*_*n*_ - wound area during distinct check-point)
- total wound area decrease per treatment time (calculated as (*A*_0_ − *A*_*n*_)/*t*, where *t* stands for healing time given in weeks
- total percent of healed area (described as: $\left(1-\frac{A_n}{A_0}\right)\ast 100\%$).
- percent of healed area per week $\left[\left(1-\frac{A_n}{A_0}\right)\ast 100\%\right]/t$,
- wound circumference to wound area ratio (C/A)

The listed parameters give a complementary picture of the dynamics of wound healing. The percentage of tissue healed ignores differences in the initial size of individual wounds, which allows the rate of healing for wounds of different sizes to be compared. In turn, determining the ratio of volume to wound area allows to take into account the shape of the wound; wounds with a circular contour will have a lower C/A ratio than those with an irregular shape. A larger volume-to-area ratio will be a favorable parameter and will characterize faster healing wounds. Data for analysis were collected during each visit; in statistical analysis, each visit is treated as a distinct case.

Several patients had more than one wound; parameters of each wound were calculated separately, so their number is greater than the number of patients.

Graphs showing the change of the above-mentioned parameters over time were created with the EpiDisplay [30] package of the R [31] program. Normality of results were determined by Shapiro-Wilk test, difference between control and treatment groups were evaluated with Student T test or non-parametrical Mann-Whitney U test, depending on the normality of results.

### Immunohistochemical reaction

The tissue sections sampled from the wounds were fixed in 10% buffered formalin, dehydrated and paraffin-embedded. The paraffin block material was cut into 4 μm thick paraffin sections and transferred on Superfrost Plus slides (Menzel Gläser, Germany). Deparaffinization, hydration and epitope exposure were then performed by boiling at 97 °C for 20 min in a Dako PT Link apparatus (Dako, Denmark) in Target Retrieval Solution buffer with an antibody specific pH according to the 3-in-1 procedure. In order to assess the intensity of the expression of the Ki-67, antibody supplied by Dako (Denmark) was used (1:100 dilution).

The IHC reaction was performed using the Autostainer PT Link 48 (Dako, Denmark) and the EnVision™ FLEX, Link visualization system (Dako, Denmark).

Ki-67 antigen expression analysis was performed using a BX41 light microscope (Olympus, Japan) under a magnification of × 200. Positive immunohistochemical reaction was observed and scored – 1-10% of positive cells – score 1; 11–25% - score 2; 26–50% - score 3; over 50% - score 4, expression was assessed on whole slide. The evaluation was carried out independently by two observers, discrepant results were reevaluated by both of them. Nondiagnostic samples (e.g. with too little epidermis or poorly fixed) were excluded from analysis.

## Results

### Dynamics of wound healing

The initial wound parameters (area, circumference, circumference/area ratio) are given in Table 2. There was no statistically significant difference between control and experimental group.

**Table 2:** Basic parameters of wounds in the experimental and control groups during the first day of treatment. No statistically significant differences were found in by the Student’s T test)

Variable	Control (SD)	Experimnental (SD)	p-value	Valid N Control	Valid N Study
Area [cm2]	15.69 (24.78)	22.40 (31.32)	0.532	15	13
Circ. [cm]	14.33 (14.78)	23.61 (25.38)	0.239	15	13
Circ./Area	2.85 (2.67)	1.88 (1.09)	0.232	15	13

**Fig. 1:**
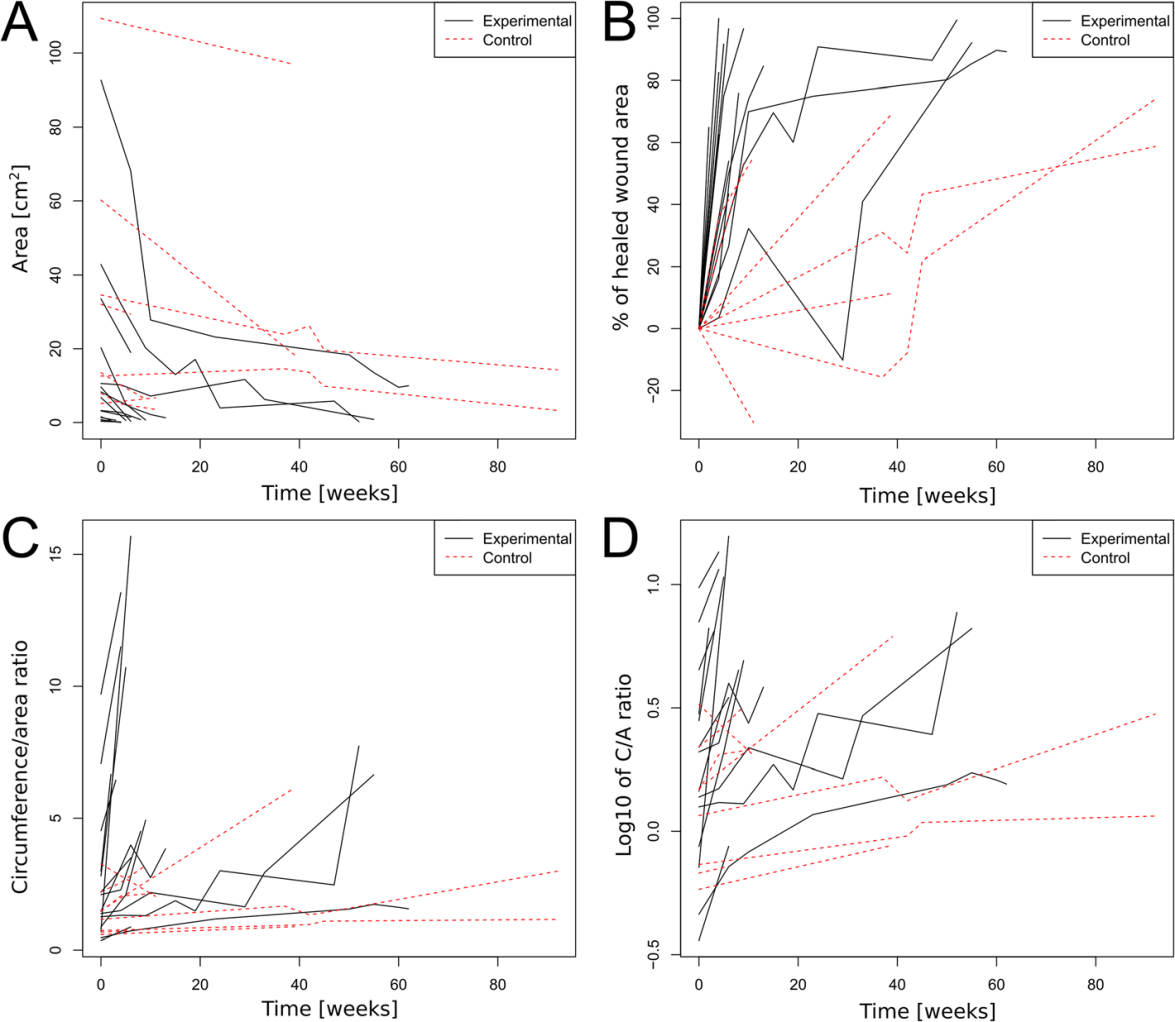
The graphs show the parameters of the examined wounds changing with time. Each line represents a single wound, and each bend corresponds with check-points. **A** - represents the wound area, **B** - wound reduction percentage (related to its initial area), **C -** circumference/area ratio (high C/A ratio represent irregular wound circumference and better healing rate) **D** - decimal logarithm of the C/A ratio, used to increase the readability of the results presented in diagram **C**

**Fig. 2:**
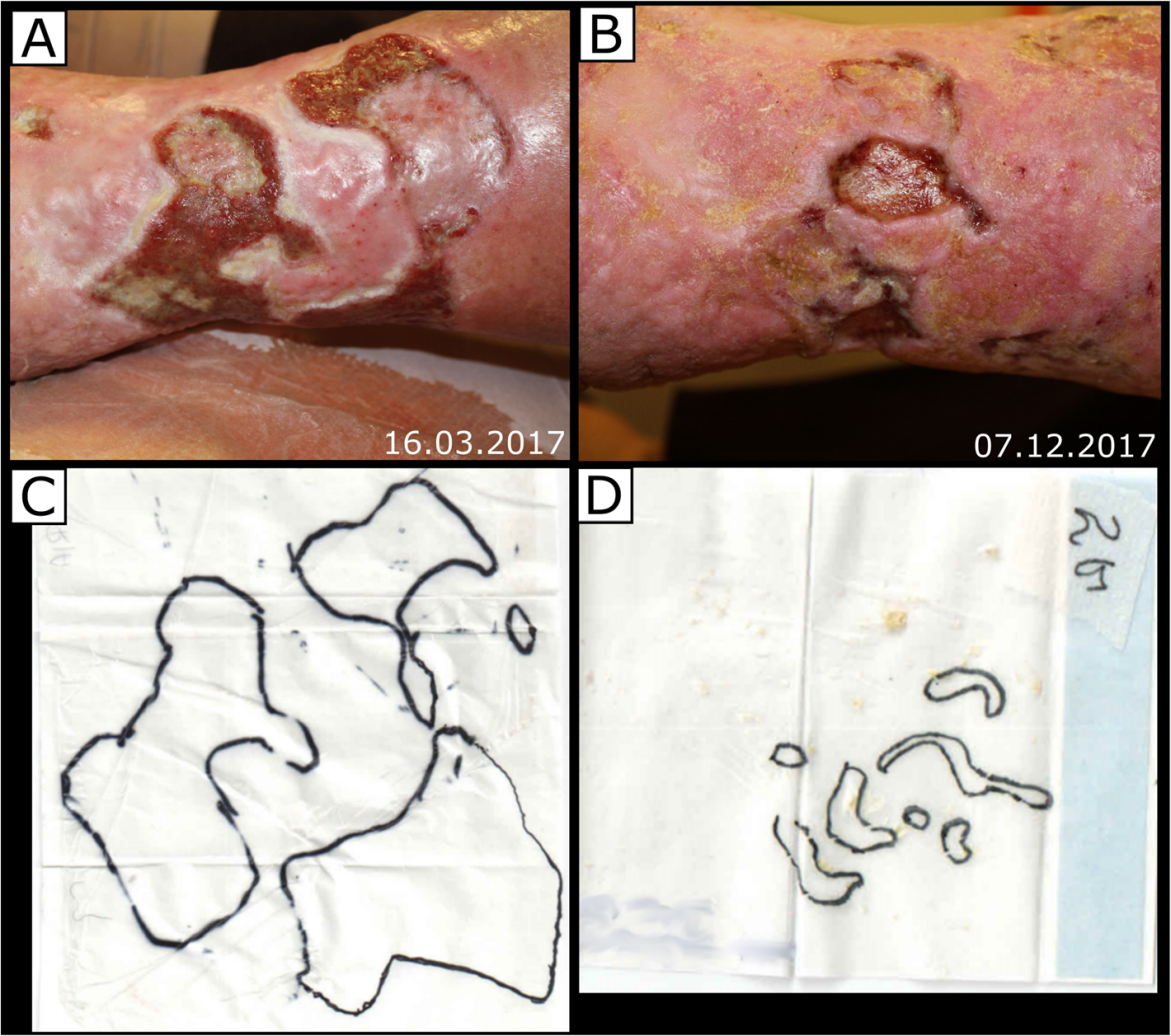
An exemplary wound treated with ASC. **A** - during treatment, **B** - after 9 months. **C** and **D** are the contours used to calculate the area and circumference of the wounds. The wound area was significantly reduced in a relatively short time

The wound healing process over time is illustrated in Fig. 1, an exemplary Revitacell treated wound is shown in Fig. 2. During the study, no side effects were observed on the wounds. The wound healing parameters were compared in Table 3. A number of statistically significant differences were found between the control and experimental groups.

**Table 3:** Statistical comparison of wound healing parameters from all valid check points – each checkpoint was treated as a distinct case, thus valid cases exceed number of patients and wounds. Due to the lack of a normal distribution in some parameters, the non-parametric Mann-Whitney U test was used. Statistically significant results are bolded

Variable	*p*-value	Experimental group Valid N	Control group Valid N
Area [cm2]	**0.009**	50	29
Circumference [cm]	**0.007**	50	29
C/A ratio	**0.027**	49	29
Wound decrease from last visit [cm^2^]	0.279	50	29
Total wound decrease [cm^2^]	**0.030**	50	29
Total wound decrease/healing time [cm^2^/weeks]	**0.005**	50	29
Total % of healed area	**0.001**	50	29
Total % of healer area/healing time [1/weeks]	**0.001**	50	29
Ki-67 score (basal layer only)	0.448	26	9
Ki-67 score (whole epidermis)	0.894	29	8

### Evaluation of Ki-67 expression

**Fig. 3:**
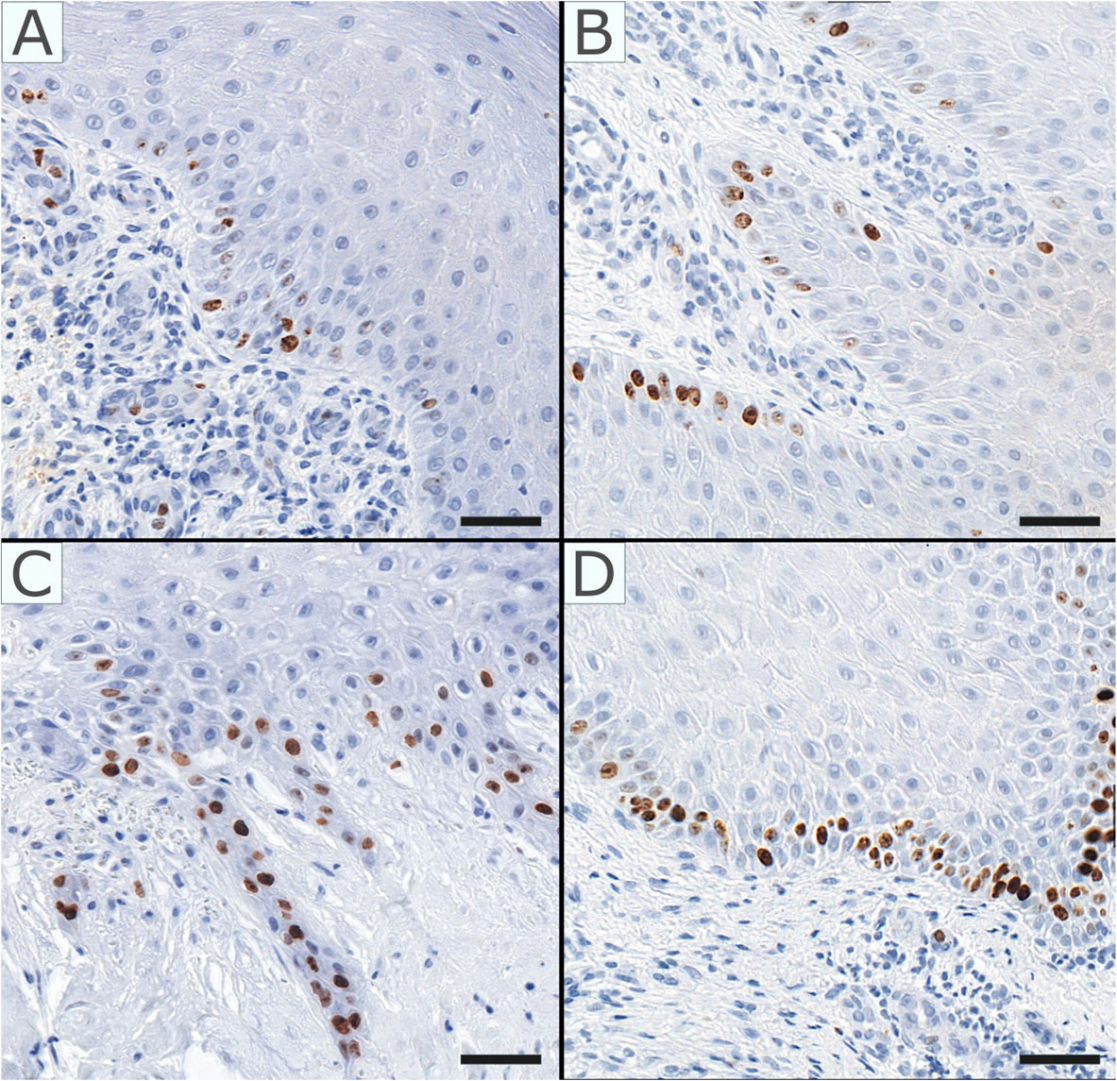
Representative histological slides with immunohistochemical staining for Ki-67. **A**: score 1 (1–10% of Ki-67 positive cells). **B**: score 2 (11–25% positive) **C**: score 3 (26–50% positive). **D**: score 4 (over 50% positive cells). Scale bar = 50 μm

The evaluation of Ki-67 positive cells in the experimental and control group is presented in Table 3, representative images are shown on Fig. 3. No statistically significant differences were obtained, regardless of whether only the basal layer or the entire epidermis were taken into account.

## Discussion

Medications containing the ASC homogenate have already been tested multiple times on animals [25–29] and have already been applied in regenerative medicine. Yet, with humans, ASC homogenate and its derivatives to date has only been used as a cosmetic [32].

Studies on a canine model using a purified form of the ASC homogenate showed a significant improvement in the biophysical properties of the skin in the healing regions; moreover, in experimental group there was significantly better hydration of the stratum corneum than in placebo group (glycerol and PBS solution) or in group without any treatment [26]. In rabbits, MIC-1 cells were used to regenerate auricular cartilage [33] and bone tissue [27]. All of the above-mentioned application studies, regardless of the route of administration, did not show any side effects or cytotoxicity.

To the best of our knowledge, current study is the first regarding medical application of ASC in human. The obtained results confirmed that the use of ASC extract has a beneficial effect on the healing process of chronic wounds. Selected statistical parameters describe the process of wound healing from the beginning of treatment to the end of the study. Wound healing is a dynamic process that changes over time, especially in the case of chronic wounds. Statistics include absolute values (change in area and perimeter), relative values (percent of wound reduction, C/A ratio) as well as changes in these values over time. Regardless of the selected parameters, they are more favorable in the test group than in the control group treated with standard procedures.

The only parameter that gave no significant statistical difference is the percentage of Ki-67 positive cells. Lack of this correlation may result directly from the methodology used; for the study, samples were taken from healing wound margins where proliferation progressed independently of the group.

As shown by Safferling et al., Ki-67 expression in wounded and undisturbed epithelium does not differ significantly; in the study, in regenerating epithelium there were from 20 to 50% Ki-67 positive cells vs baseline 15% of positive cells in normal epidermis [34]. Obtaining statistically significant results between two differently treated chronic wound would require precise positive cell counting and bigger population of the study – or more pronounced differences in proliferation rate. In the study by Noguchi et al. evaluation of Ki-67 in epidermis successfully demonstrated the difference in wound healing process between wild type and MED1-null mice, however, the difference in Ki-67 positive cells was tenfold [35]. Such differences are rather unlikely in two almost equally treated groups.

In the present study, the level of Ki-67 did not correlate with any of the wound healing parameters (R < 0.2; p > > 0.05 with Spearman correlation test, data not shown), so it does not faithfully reflect the speed of wound healing. Lack of significant statistical differences in its expression does not contradict with other results.

Due to the small size of the population of study, the obtained results should be treated as preliminary - yet promising. Results correspond to those obtained in similar studies, also using stem cells in wound healing [36]. The mechanism of action of ASC extract remains to be elucidated. As there was no living ASC in the sonicated extract, regeneration was presumably supported by proteins and other components produced beforehand by living cells, similarly as in therapy with Grafix (containing dehydrated and cryopreserved cells) [23, 32].

It is especially important within the context of bioethics, since using living animal cells for therapies in human raises major ethical and/or legal concerns [37]. Even if xenotransplantation of living antlerogenic stem cells showed some potential in animal studies [27], it would be definitely controversial to apply such therapy in human. Using dead cells and/or animal products in therapies is commonly used [38] and may be considered far more acceptable than xenotransplantation – even if the product is derived from cell culture, not from the living animal. Another major concern is the safety of using ASCs homogenate. However, to the best of our knowledge, no undesirable side effects were reported up to date [25–29, 32].

Future research may focus on both repeating the study using a larger group of patients, and trying to elucidate the mechanism of action of Revitacell.

## Data Availability

The datasets used and/or analyzed during the current study are available from the corresponding author on reasonable request.

## Abbreviations

ASC: Antlerogenic Stem Cells
ECM: Extracellular Matrix
MSCs: Mesenchymal Stem Cells
BM-MSCs: Bone Marrow Derived-MSCs
ASCSs: Adipose Derived MSCs
C/A: wound Circumference to Area ratio
